# Effect-Directed Analysis for the Antioxidant Compound in *Salvia verticillata*

**Published:** 2016

**Authors:** Bahman Nickavar, Javad Rezaee, Azar Nickavar

**Affiliations:** a *Department of Pharmacognosy, School of Pharmacy, Shahid Beheshti University of Medical Sciences, Tehran, Iran. *; b *Aliasghar Children’s Hospital, Iran University of Medical Sciences, Tehran, Iran.*

**Keywords:** Antioxidant activity, Chrysoeriol, Labiatae, * Salvia verticillata*, TLC-DPPH bioautography analysis

## Abstract

*Salvia* genus is one of the largest genera of the Lamiaceae family. Its species have been used for a wide variety of disorders in the local traditional medicine systems. Therefore, the genus has been the subject of several phytochemical and biological studies. The aim of the study was to identify the major antioxidant compound(s) from the methanol extract of *Salvia verticillata* using activity-guided fractionation. The crude extract showed strong antioxidant activities in DPPH and β-carotene/linoleic acid tests. The ethyl acetate fraction also exhibited a potent free radical scavenging activity compared to the other fractions. Further fractionation and purification of the ethyl acetate fraction using chromatography methods yielded a compound with high antioxidant capacity. The isolated active compound was determined as chrysoeriol. It showed a dose-dependent free radical scavenging activity with an IC_50__(DPPH scavenging)_ value of 93.32 (80.23 – 108.57) mM.

## Introduction

Recently, the role of reactive oxygen species (ROS) in biological systems and pathological conditions has been in the focus of research interests. ROS are the major free radicals (FRs) in human body that are considered to be the prim causes of oxidative damage (1). They can attack cellular biomolecules such as lipids, proteins and DNA, leading to various chronic disorders e.g. neurological disorders, cancer, diabetes, inflammation, etc. (2). FRs can also induce oxidation of lipids in fat-based foods, affecting the quality and acceptability of the products (3). 

The main task of the antioxidants is defense of organisms and/or food and pharmaceutical products from the harmful effects of FRs. Generally, antioxidants are able to scavenge FRs, and therefore to inhibit or retard the oxidation process (4). Synthetic antioxidants such as BHT, BHA, and TBHQ are commonly used in the food industry to delay the oxidation degradation and to improve the shelf life (5). However, the safety of the synthetic antioxidants has been questioned and some restrictions have been placed on their application (6). For the reason, there is a growing interest in identifying the effective and safe natural antioxidants and several natural sources are being examined (2, 7). Various plant-derived natural compounds such as polyphenols and terpenoids have been found to act as antioxidants and free radical scavengers (8, 9). 

The plants of the Lamiaceae family are regarded as an important source for natural antioxidants. For instance, sage (*Salvia officinalis*) and rosemary (*Rosmarinus officinalis*) are well known for their potent antioxidant properties (10). *Salvia* L. is one of the largest genera of the Lamiaceae family with over 1000 species worldwide. Some members of the genus are used as culinary herb, spice, tea and in perfume and cosmetic industries. Also, some *Salvia* species have been used in folk medicine because of their diverse biological activities. Phytochemical investigations have shown that *Salvia* taxa are mainly rich in terpenoids and phenolic compounds (11-13).

Iran is an important country for *Salvia* species in the world. The flora of Iran includes 58 species of the genus (14). However, the majority of *Salvia* species growing wild in Iran have not been evaluated from phytochemical and pharmacological point of view. In the research for new antioxidant sources, we have studied the antioxidant capacity of some *Salvia* species from Iran and have found that *Salvia verticillata* has high antioxidant activities (15). Other researchers have also shown the prominent antioxidant activities of the species (16). Phytochemical analysis have revealed the presence of volatile constituents (17-22), ditrpenoids (23, 24), triterpenoids (23, 25), phenolic acids (25, 26) and flavonoids (25, 27) in different parts of *Salvia verticillata*. In the present work, an activity-guided isolation was carried out to identify the major antioxidant compound(s) from aerial parts of *Salvia verticillata*.

## Experimental

*General*

^1^H and ^13^C NMR spectra were recorded in DMSO-*d6 *using TMS as internal standard on a Varian 400 spectrometer at 400 MHz and 100 MHz, respectively. EI-MS spectra were obtained on a Finigan-Mat spectrometer (70 ev). UV-Vis spectra were recorded on a Shimadzu Multispect-1501 UV-Vis spectrophotometer in methanol. 

All chemicals used in this study were purchased from Sigma-Aldrich Chemical Co. (France) or Merck Company (Germany). 

*Plant material*

*Salvia verticillata* L. was collected from Tehran province during the flowering period in summer 2012 and authenticated by M. Kamalinejad at the Herbarium of the Department of Pharmacognosy, School of Pharmacy, Shahid Beheshti University of Medical Sciences where the voucher specimens (No. 1072) have been preserved. The plant was dried at ambient temperature with active ventilation. 

*Extraction, fractionation and isolation*

The air-dried aerial parts of the plant (250 g) were extracted thrice using 80% methanol by maceration method. The supernatant was filtered and concentrated *in vacuo *to give the crude extract (27.8 g). 

The crude extract (25 g) was suspended in water and then partitioned successively in different solvents, namely *n*-hexane (HX), chloroform (CF), ethyl acetate (EA), and water (W). The fractions were filtered and concentrated by rotary evaporator. 

Since the EA fraction showed the highest DPPH (1,1'-diphenyl-2-picrylhydrazyl) radical scavenging activity among the other fractions, it was further fractionated using a silica gel 60 column (70 – 230 mesh) by a chloroform/ethyl acetate step-gradient elution. The collected fractions (F_1_ – F_11_) were evaluated by DPPH test. Fraction F_8_, which elicited radical scavenging activity, was submitted to repeated preparative layer chromatography on coated plates with silica gel 60F_254_ (230 – 400 mesh) using CHCl_3_/EtOAC/HCOOH (45:45:10, v/v/v) as the best developing system. The bands that showed DPPH scavenging activity were scraped off, eluted by ethyl acetate, and monitored by TLC on precoated plates with silica gel 60F_254_. Further purification and recrystallisation resulted in the active compound. The pure compound was identified by ^1^H, ^13^C NMR, MS spectra and by comparison with the literature data. 

*Antioxidant assays*

*DPPH radical scavenging activity*

The free radical scavenging activities of the crude extract, fractions and the pure compound were evaluated by measuring their ability to scavenging DPPH radicals. 

The preliminary test was performed with a rapid TLC scavenging technique (TLC-bioautography analysis) according to a modified method described by Nickavar *et al*., 2014 (28). An aliquot of each sample (5 μL, MeOH) was directly deposited onto the TLC plate (silica gel 60F_254_ TLC plates) and developed in CHCl_3_/EtOAC/HCOOH (45:45:10, v/v/v). The developed plate was allowed to air-dry and followed by spraying with a DPPH solution (0.2%, MeOH). 30 min later, active compounds appeared as yellow spots against a purple background. Gallic acid was used as the positive control. 

The spectrophotometric assay was carried out according to the method explained by Nickavar and Esbati, 2012 with some modifications (29). An aliquot of each sample (200 μL, MeOH) was mixed with 2 mL of a 0.1 mM DPPH solution in MeOH. After 30 min, the absorbance of each sample was recorded at 517 nm. Different concentrations were prepared for each sample and analyzed in triplicate. The percentage of scavenged DPPH was calculated according to the following equation (Equation 1):

$I_{\mathrm{DPPH}}\left(\%\right)=100.\left[\frac{A_{\mathrm{control}}-(A_{\mathrm{sample}}-A_{\mathrm{blank}}}{A_{\mathrm{control}}}\right]$

Equation 1

Where A_C_ is the absorbance of the control (200 μL of MeOH with 2 mL DPPH solution), A_B_ is the absorbance of the blank (200 μL sample with 2 mL MeOH) and A_S_ is the absorbance of the sample. 

*β-Carotene/linoleic acid bleaching assay*

Antioxidant activities of the crude extract, fractions and the pure compound were evaluated using β-carotene/linoleic acid bleaching model system as described by Nickavar and Esbati, 2012 with some modifications (29). 2 mL of β-carotene (200 μg/mL, CHCl_3_) was added to a flask containing linoleic acid (45 mg) and tween-40 (400 mg). Chloroform was evaporated under a stream of nitrogen. 100 mL of distilled water saturated with oxygen was added and shaken vigorously. 0.5 mL of different concentrations of each sample in methanol was transferred into different test tubes containing 4.5 mL of the above mixture. As soon as the emulsion was added to each tube, the zero time absorbance was measured at 470 nm using a spectrophotometer. The samples were then subjected to thermal autoxidation by keeping them in a constant temperature water bath at 50 °C for 2 h. Subsequently absorbance values were recorded after incubation. The antioxidant activity was calculated according to the following equation (Equation 2): 

$I_{\mathrm{bleaching}}\left(\%\right)=(\mathrm{Absorbance of sample after 2 h}/\mathrm{Initial absorbance of sample})\times \mathrm{100}$

Equation 2

*Statistical analysis*

The IC_50_ values were calculated from logarithmic regression curves (I% against sample concentration) with normalized data and presented with their respective 95% confidence limits. The assays were performed in triplicate. All the statistical analysis was accomplished using the computer software GraphPad prism 3.02 for windows (GraphPad Software, San Diego, CA, USA).

## Results and Discussion

**Figure 1:**
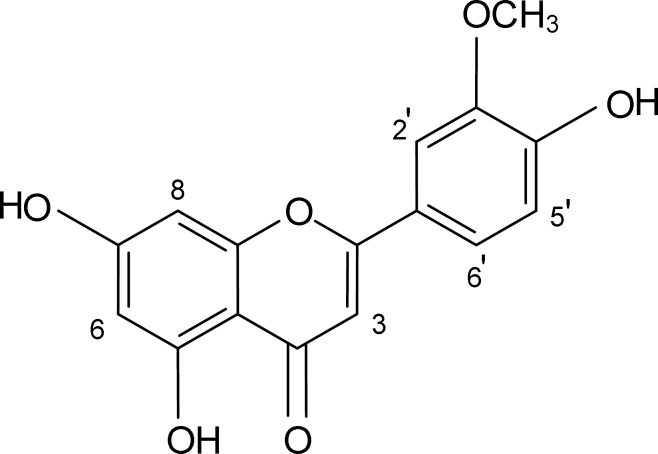
Chemical structure of chrysoeriol

The conventional antioxidant tests were first carried out to determine the antioxidant capacity of the crude extract of *Salvia verticillata*. The crude extract exhibited remarkable scavenging capacity toward DPPH radical and strong inhibitory effect on bleaching of β-carotene in a concentration-dependent manner with IC_50_ values of 134.10 (114.80 – 156.70) mcg/mL and 194.00 (170.40 – 220.90) mcg/mL, respectively (Table 1). In order to separate the active components presented in the crude extract, solvent fractionation was performed with *n*-hexane, chloroform, ethyl acetate and water, successively. As shown in Table 1, the DPPH radical was significantly scavenged by the ethyl acetate fraction with an IC_50_ value of 23.89 (20.61 – 27.68) mcg/mL. The fraction was monitored by the TLC-DPPH method and at least two bands were observed to possess high scavenging activity. Therefore, it was fractionated for further purification by chromatographic techniques. Finally, a pure active compound (Figure 1) was isolated. The compound was obtained as a yellow amorphous powder with R_f_ = 0.72 on TLC plate (silica gel 60F_254_) with CHCl_3_/EtOAC/HCOOH (45:45:10, v/v/v) solvent system. The spectral characteristics of the compound were as follows:

^1^H NMR (400 MHz, in DMSO-*d6*), δ: 3.88 (s, 3H, OCH_3_-3'), 6.12 (br. s, 1H, H-6), 6.40 (br. s, 1H, H-8), 6.54 (s, 1H, H-3), 6.83 (d, *J* = 8.1Hz, 1H, H-5'), 7.32 (br. s, 1H, H-2'), 7.36 (d, *J* = 8.1 Hz, 1H, H-6').

^13^C NMR (100 MHz, in DMSO-*d6*), δ: 54.2 (OCH_3_), 92.7 (C-8), 97.6 (C-6), 101.6 (C-3), 102.3 (C-10), 110.1 (C-2'), 115.2 (C-5'), 120.2 (C-6'), 129.8 (C-1'), 145.6 (C-3'), 148.4 (C-4'), 153.8 (C-2), 155.1 (C-9), 160.9 (C-5), 167.1 (C-7), 170.5 (C-4).

EI-MS (70 ev), *m/z* (*I%*): 300 (12%), 286 (82%), 153 (27%), 151 (22%).

UV-Vis: λ_max_ (in CH_3_OH) = 267 (sh.), 356 nm.

Detailed analysis of the spectral data showed that the compound was chrysoeriol and all of data matched with those reported in the literature (2, 30). It scavenged DPPH radicals in a dose dependent manner and the IC_50_ valve was 93.32 (80.23 – 108.57) mM.

Phenolic compounds, particularly flavonoids, of the genus *Salvia* have received much attention due to their relevant biological properties (31). Chrysoeriol has already been isolated from some *Salvia* species such as *Salvia candidissima* and *Salvia palaestina* (32, 33). However, to the best of our knowledge, this is the first report on the isolation of chrysoeriol from *Salvia verticillata* and its antioxidant activity. 

**Table 1:** Antioxidant activities and IC_50_ values of the crude extract and fractions of *Salvia verticillata* and its active compound chrysoeriol

**Sample**	**IC**_50__(DPPH scavenging)_	**IC**_50__(β-Carotene bleaching)_
Crude extract	134.10 (114.80 – 156.70) [mcg/mL]	194.00 (170.40 – 220.90) [mcg/mL]
HX fraction	NA^a^	-
CF fraction	NA^a^	-
EA fraction	23.89 (20.61 – 27.68) [mcg/mL]	-
H_2_O fraction	46.48 (41.65 – 51.87) [mcg/mL]	-
Rutin	45.99 (38.74 – 54.66) mM	NA^a^
Vitamin C	135.70 (112.42 – 163.81) mM	NA^a^
Gallic acid	75.00 (67.83 – 82.88) mM	1091.58 (952.27 – 1252.06) mM
Chrysoeriol	93.32 (80.23 – 108.57) mM	561.18 (518.22 – 607.81) mM

a NA: not active
